# Midgut Volvulus in Disguise: Acute Abdomen in Early Pregnancy

**DOI:** 10.7759/cureus.50955

**Published:** 2023-12-22

**Authors:** Khalid Gashoot, Muataz Omar Kashbour, Maaly Abuhlaiga

**Affiliations:** 1 Diagnostic Radiology, Tripoli Central Hospital, Tripoli, LBY; 2 Radiology, Tripoli University, Tripoli, LBY; 3 Diagnostic Radiology, National Cancer Institute, Misrata, LBY; 4 Internal Medicine, Misrata Medical Center, Misrata, LBY

**Keywords:** midgut volvulus, magnetic resonance imaging, intestinal volvulus, small bowel obstruction, pregnancy

## Abstract

Small bowel obstruction (SBO) in pregnancy is a challenging diagnosis. Case rarity, non-specific presentations, and the non-practicality of using X-ray/gamma-ray imaging modalities in pregnancy contribute to the increased difficulty in timely diagnosing midgut volvulus during pregnancy, thereby increasing maternal and fetal morbidity. We report a case of midgut volvulus in a 39-year-old lady, gravida 3, para 2, with two previous cesarean sections. Her only presenting complaint was abdominal pain for three days with no other associated symptoms. The case was successfully diagnosed using magnetic resonance imaging (MRI) and subsequently treated surgically by segmental resection with side-to-side ileocecal anastomosis, thereby saving the mother and fetus. Clinicians should have a low threshold of suspicion of the varied causes of SBO in pregnancy, particularly in patients with prior abdominopelvic surgeries. Imaging is central to preoperative diagnosis, and MRI has gained popularity with safety and accuracy comparable to computed tomography. Management aims at minimizing maternal and fetal morbidity and mortality.

## Introduction

Intestinal obstruction in pregnancy is uncommon, with incidence ranging from 1 in 1,500 to 1 in 66,000 births, and constitutes the second most common non-obstetric cause requiring surgical intervention in pregnancy [1,2]. Small bowel obstruction (SBO) in pregnancy is a rare challenging diagnosis, with a reported incidence of 1 in 17,000 deliveries [3]. Case rarity, non-specific presentations, and the contraindication of X-ray/gamma-ray imaging modalities in pregnancy contribute to the increased difficulty in timely diagnosing SBO during pregnancy, thereby significantly increasing maternal (2% to 4%) and fetal (13 to 17%) mortality [3,4]. Here, we report the successful detection and management of a case of small bowel volvulus in a pregnant lady with prior pelvic surgery utilizing magnetic resonance imaging (MRI).

## Case presentation

A 39-year-old Libyan lady, gravida 3, para 2, live 2, with previous two cesarean sections, presented to the emergency department of Sebrata Clinic at eight weeks of gestation complaining of diffuse abdominal pain for the past three days with no other associated symptoms. There was no past medical history.

Upon presentation, all vitals were stable, and her general examination was within normal limits. On abdominal examination, there was noted distension, generalized abdominal pain and diffuse tenderness, and reduced bowel sounds, but no organomegaly. Obstetric examination was normal. Laboratory evaluation of her complete blood count revealed a normal leukocyte count with marginally raised neutrophils and marginally lowered lymphocytes. Electrolytes and kidney and liver functions were normal. However, C-reactive protein (11 mg/dL), procalcitonin (24.4 ng/mL), and lactate (29.72 mg/dL) were elevated (Table 1).

**Table 1:** Laboratory results. CBC: complete blood count; WBC: white blood cell; MCV: mean corpuscular volume; MCH: mean corpuscular hemoglobin; ALT: alanine aminotransferase; AST: aspartate aminotransferase; ALP: alkaline phosphatase; PCT: procalcitonin; CRP: C-reactive protein

CBC		Reference range
WBC	8.27 × 10^3^/µL	3.5–9.5 × 10^3^/µL
Neutrophils	6.8 × 10^3^/µL	1.8–6.3 × 10^3^/µL
Lymphocyte	0.94 × 10^3^/µL	1.1–3.2 × 10^3^/µL
Monocyte	0.36 × 10^3^/µL	0.1–0.6 × 10^3^/µL
Eosinophils	0.09 × 10^3^/µL	0.02–0.52 × 10^3^/µL
Basophils	0.02 × 10^3^/µL	0.00–0.06 × 10^3^/µL
RBC	4.03 × 10^6^/µL	3.8–5.8 × 10^6^/µL
Hemoglobulin	12.5 g/dL	11.5–17.5 g/dL
HCT	35.8%	35–50%
MCV	88.5 fL	82–100 fL
MCH	31 pg	27–34 pg
PLT	239 × 10^3^/µL	125–350 × 10^3^/µL
Biochemistry		
Creatinine	0.6 mg/dL	0.4–1.2 mg/dL
Urea	17 mg/dL	0–50 mg/dL
Sodium	138 mEq/L	135–145 mEq/L
Potassium	3.8 mEq/L	3.5–5 mEq/L
Chloride	98 mEq/L	95–105 mEq/L
ALP	42 U/L	35–139 U/L
AST	24 U/L	0–40 U/L
ALT	10 U/L	0–41 U/L
Bilirubin total	0.8 mg/dL	0–1.2 mg/dL
PCT	24.4 ng/mL	<0.50 ng/mL
Glucose	125 mg/dL	70–125 mg/dL
Lactate	29.72 mg/dL	4.9–19.8 mg/dL
CRP	11 mg/L	0–6 mg/L

**Figure 1:**
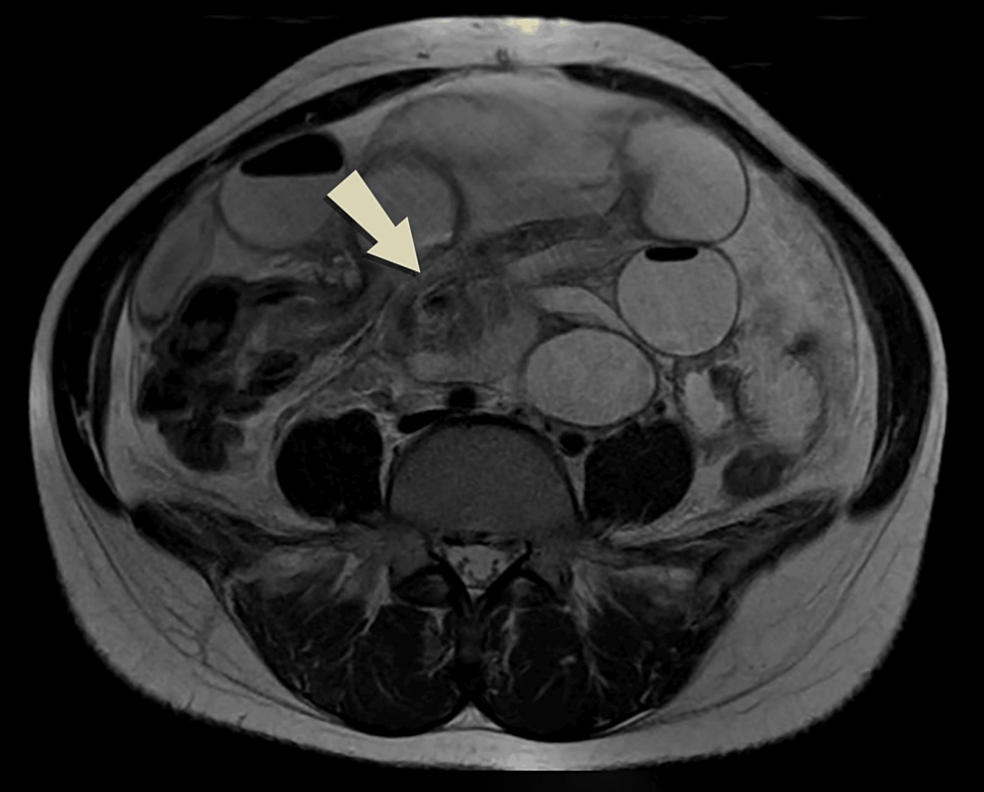
Axial T2 imaging shows a small bowel dilated with air-fluid levels and abnormal C-shaped configuration with twisting of the mesentery giving a whirlpool sign.

**Figure 2:**
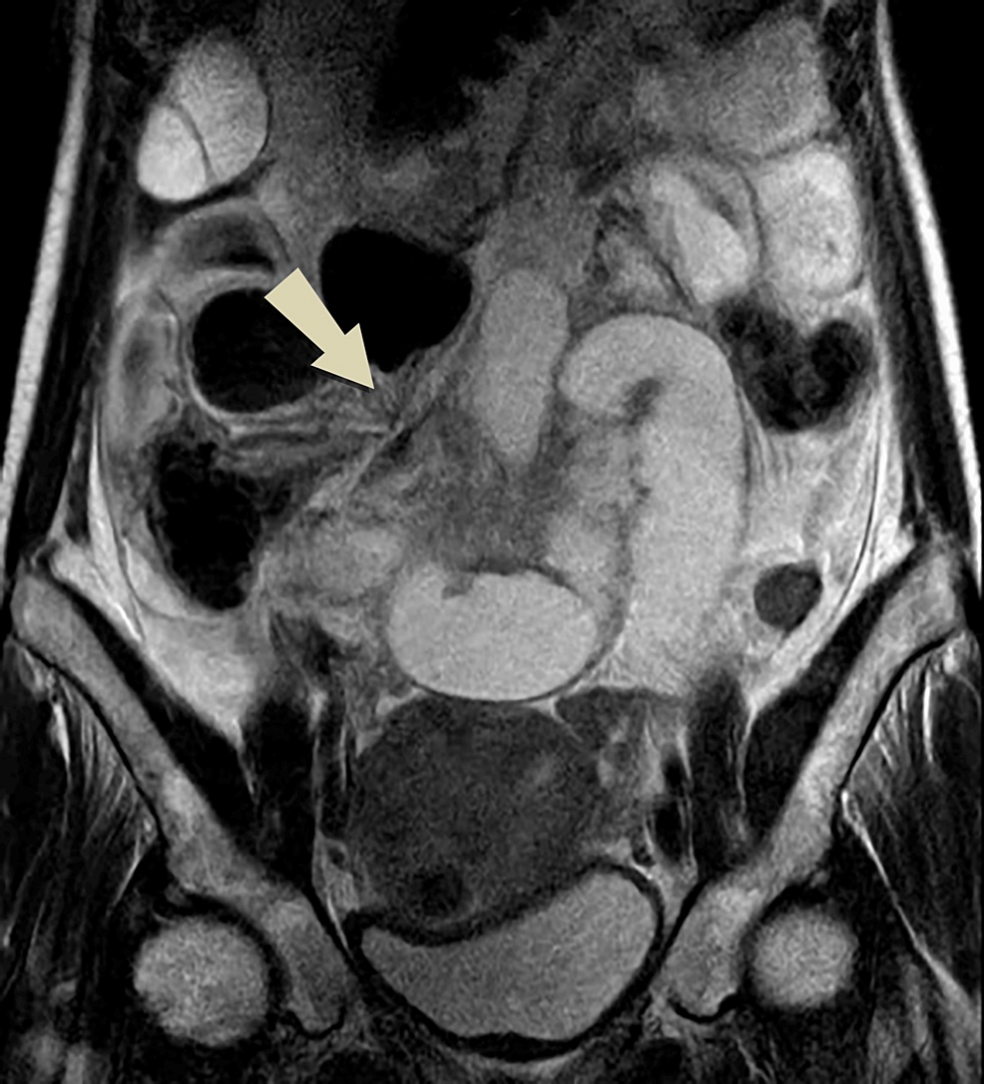
Coronal T2 imaging shows a dilated small bowel with abnormal configuration and mesenteric twisting.

**Figure 3:**
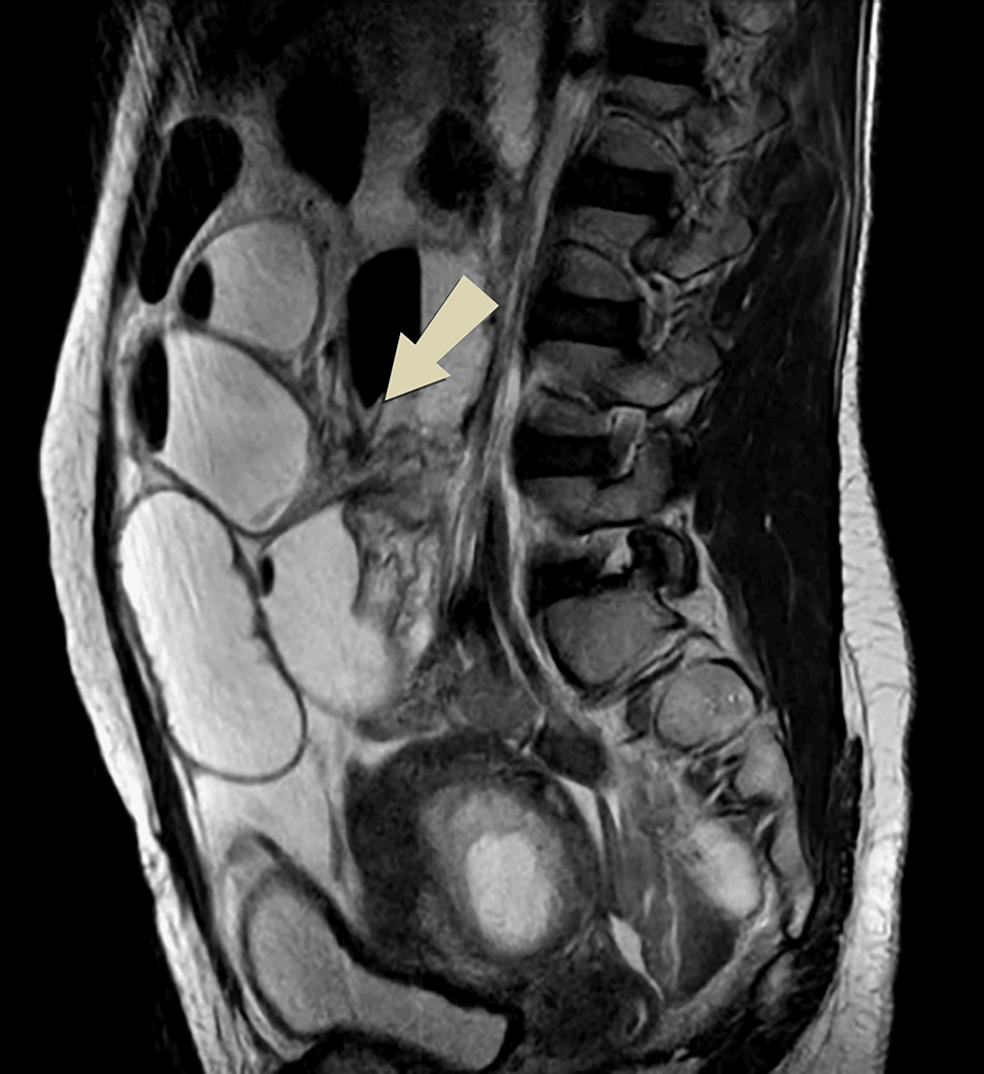
Sagittal T2 imaging shows a dilated small bowel with air-fluid level intensity seen arranged in an abnormal configuration and mesenteric twisting.

Ultrasonography of the abdomen and pelvis demonstrated a single intrauterine viable gestational sac, along with dilated small bowel loops with no peristaltic movement suggesting SBO. The patient was admitted to the surgical ward and a non-contrasted MRI of the abdomen and pelvis was arranged. The MRI revealed a small bowel dilated with the air-fluid level intensity seen arranged in an abnormal C-shaped configuration in the center of the abdomen with twisting of its mesentery giving a whirlpool sign. Moderate ascites was also noted (Figures 1-3).

The surgical team performed an exploratory laparotomy with midline locating the gangrenous twisted ileum. Segmental resection with side-to-side ileocecal anastomosis was done. Postoperative care progressed smoothly, and after four days, the patient was discharged in a healthy condition while preserving her pregnancy.

## Discussion

Acute abdominal pain in pregnancy is a complex complaint as pregnancy causes unusual presentations for common differential diagnoses of abdominal pain, and the differential list varies with gestational age, further complicating prompt diagnosis. For example, ectopic pregnancy is a common cause of abdominal pain in the first trimester, whereas preterm labor and placental abruption are common in late pregnancy. Acute appendicitis, acute cholecystitis, gastroenteritis, and pancreatitis are common causes of abdominal pain that may present during pregnancy [3].

Adhesions from prior abdominal surgeries are the most common etiology for mechanical SBO, accounting for 60-70% of cases. The remaining cases are due to volvulus (25%) and intussusception (5%) [1,2,4]. The incidences during the first, second, and third trimesters and puerperium are reported as 6%, 28%, 45%, and 21%, respectively [1,2]. Adhesive obstructions are more common with open surgeries compared to laparoscopic surgeries. Volvulus, being the second most common cause, is a closed-loop mechanical bowel obstruction caused by abnormal twisting of a bowel loop more than 180 degrees around the axis of its own mesentery [5].

Common presenting features of SBO in pregnancy include abdominal pain, vomiting, and constipation, with abdominal tenderness detected on examination [1,2,6]. A systematic review published in 2014 reviewing the literature on SBO in pregnancy reviewed the various presentations of pregnant women with SBO. Presenting symptoms included abdominal pain (88%) and vomiting (67%), and examination findings included tenderness (49%) and distension (28%) [4,7]. Laboratory investigations frequently returned normal. The goal of care in SBO is prompt recognition and surgical intervention, if needed, to save the threatened bowels. Confirming the diagnosis relies on the clinician’s suspicion, as well as the timely use of proper imaging.

Plain abdomen X-rays have low sensitivity during pregnancy and are generally avoided. Ultrasound may demonstrate fluid-filled bowel loops indicative of obstruction, as was seen in our patient. Ultrasound may aid in assessing intestinal peristalsis and blood flow and serves a vital role in excluding common abdominal pathologies [2]. Computed tomography (CT) is avoided in pregnancy as the risk of fetal radiation is high [5]. Imaging should, therefore, proceed to MRI where available. MRI has become an important tool for the evaluation of acute abdomen in pregnancy as it offers high soft tissue resolution enabling radiologists to identify intraluminal or extraluminal obstructions and the subsequent need for surgery without the need for a contrast agent [1]. Unal et al. reviewed 20 cases of acute abdominal pain in pregnancy and revealed that ultrasonography was compatible with intraoperative findings in 55%, on the other hand, MRI results correctly matched with intraoperative diagnoses in 83% [8]. Small bowel MRI requires special sequences to reduce motion artifacts produced by bowel peristalsis and fetal movement. A fat-suppressed true fast imaging with steady-state free precession sequence for MRI of the small bowel is generally used [9]. In complete obstruction, the bowel loops are distended with fluid and are detected easily on MRI whereas partial obstruction may pose a greater diagnostic difficulty [1]. Particular attention should be paid to pseudo-stenosis occurring due to the compression of the gravid uterus on bowel loops which leads to luminal narrowing but without surrounding inflammatory changes [10]. In our case, MRI revealed dilated small bowel loops with fluid levels seen arranged in an abnormal C-shaped configuration at the lower abdomen with twisting of its mesentery giving rise to the whirlpool sign suggestive of small bowel volvulus.

Treatment goals are centered around saving the threatened bowel with timely surgery while involving obstetricians alongside surgeons to improve outcomes. Conservative treatment should initially be attempted particularly when the cause is adhesions which can be managed conservatively, while those caused by volvulus, like our patient, mandate surgery [10]. Nasogastric aspiration and aggressive intravenous fluids to correct electrolyte disturbances are the mainstay of conservative management [2,11]. Failure of conservative measures, the detection of complete obstruction on CT, peritonitis, intractable vomiting, or the presence of signs of fetal distress are indications to proceed to surgery [2,11,12]. Prophylactic tocolytics may be administered to reduce the risk of premature labor particularly when conservative therapy fails. In pregnant women, when SBO is highly suspected, an exploratory laparotomy should be performed regardless of imaging results [6]. Therefore, a midline incision is usually needed for adequate visualization and complete exploration of the bowels with minimum manipulation of the gravid uterus [1,11].

## Conclusions

SBO during pregnancy is a rare and unexpected differential, but it is important to consider surgical causes of abdominal pain beyond obstetric or gynecological causes, including bowel obstruction, particularly in patients with previous abdominal surgeries. The non-specific presentation further complicates case detection, and, therefore, reliance on radiological investigations is crucial for rapid diagnosis, among which MRI is gaining particular popularity as a safe imaging modality during pregnancy. All efforts should be directed toward avoiding delayed diagnosis and prompt surgical intervention aiming to reduce the high morbidity and mortality rates.
